# Editorial: Analysis of performance in small-sided games in team sports

**DOI:** 10.3389/fspor.2024.1391392

**Published:** 2024-03-12

**Authors:** Henrique de Oliveira Castro, Filipe Manuel Clemente, Gibson Moreira Praça, Lorenzo Laporta, Gustavo De Conti Teixeira Costa

**Affiliations:** ^1^ Physical Education Department, Universidade Federal de Mato Grosso—UFMT, Cuiabá, Brazil; ^2^ Escola Superior Desporto e Lazer, Instituto Politécnico de Viana do Castelo, Viana do Castelo, Portugal; ^3^ Research Center in Sports Performance, Recreation, Innovation and Technology—SPRINT, Melgaço, Portugal; ^4^ Department of Biomechanics and Sport Engineering, Gdansk University of Physical Education and Sport, Gdansk, Poland; ^5^ Sports Department, Universidade Federal de Minas Gerais—UFMG, Belo Horizonte, Brazil; ^6^ Núcleo de Estudos em Performance Analysis Eportiva, Universidade Federal de Santa Maria—UFSM, Santa Maria, Brazil; ^7^ Physical Education Department, Universidade Federal de Goiás—UFG, Goiânia, Brazil

**Keywords:** small-sided games, conditioning games, team sports, game manipulation, performance analysis, sports training, sports pedagogy

**Editorial on the Research Topic** Analysis of performance in small-sided games in team sports

This Frontiers Research Topic compiles studies focused on performance analysis using small-sided games (SSGs) in team sports. Specifically, it emphasizes various aspects such as the manipulation and adaptation of new game scenarios, evaluation and training of tactical and technical variables within SSGs, assessment and monitoring of physiological, physical, and psychological variables, utilization of technology for assessing and monitoring SSG-related variables, and the application of SSGs as teaching and training strategies for sports-related aspects.

SSGs serve as pedagogical tools for teaching and training in team sports. They typically involve modifications to the playing court size, game configuration, confrontation conditions (such as numerical equality or unbalanced games), and rules, among other factors that influence training sessions. These modifications have acute and chronic effects on various aspects of sports performance, ultimately aiding in achieving the objectives intended by the head coach.

Even though the literature is consistent on the effectiveness of SSGs, there remains much to be understood about their pedagogical application in sports. In light of this gap, this Frontiers Research Topic aimed to facilitate the dissemination of knowledge in this domain, and the following section presents summaries of the published articles.

Investigating the influence of self-talk on static and dynamic three-point shot performance, Yang et al. compared the effects of quiet and noisy conditions while employing instructional self-talk, motivational self-talk, and a control group. Results revealed differences between conditions: noise significantly affected static three-point shot performance among national second-level basketball players, while dynamic three-point shot performance remained unaffected. The study suggests that self-talk interventions can effectively mitigate the negative impact of noise on static three-point shots. However, their effectiveness in tasks with high physical demands, such as dynamic three-point shots, appears somewhat limited.

Comprehending the relationship between SSGs training and match demands is crucial for designing targeted and transferable training loads. Within this context, Savolainen et al. conducted a study examining the most demanding passages of soccer matches played by national-level females and their correlation with SSGs and large-sided games (LSGs). These demanding passages were analyzed within 1, 3, or 5 min intervals, with game-based tasks involving 4-a-side (SSG) and 8-a-side (LSG) conditions. Results indicated that both SSGs and LSGs can surpass match average loads, suggesting their utility for training sessions aiming for high metabolic demands. This study reinforces the potential effectiveness of SSGs in preparing players for specific game demands, though further intervention studies are recommended to analyze chronic effects.

In Rugby, Zanin et al. investigated the association between external load (EL) and internal load (IL) variables during three SSGs involving 40 professional Rugby Union players. The SSGs designs included one for forwards (SSG-F), one for backs (SSG-B), and one for both backs and forwards (SSG-BF). IL was assessed using Stagno's TRIMP, while EL was measured using total distance, high-speed running distance, average acceleration and deceleration, PlayerLoad, PlayerLoad slow, get-up, and first-man-to-ruck variables. Results revealed associations between IL and various EL variables depending on the SSG utilized. Additionally, in the SSG-BF, IL varied between backs and forwards. The authors emphasize the necessity of manipulating different SSGs restrictions to elicit specific physiological responses, considering their playing positions.

In line with investigations on mechanical and psychophysiological workload and their correlation with neuromuscular performance and patellar tendon adaptations, Guthrie et al. aimed to assess intra-individual associations between workloads, patellar tendon properties, and neuromuscular performance in male National Collegiate volleyball athletes over the season. Results revealed associations within subjects along mechanical load-response pathways, with no such associations observed along psychophysiological load-response pathways. Furthermore, associations between subjects were identified in both load-response scenarios when neuromuscular performance was the outcome. Additionally, associations within and between subjects were observed in both load-response pathways regarding patellar tendon properties. Thus, monitoring training performance adaptations can contribute to understanding changes in tendon compositions, facilitating the management and reduction of the risk of developing patellar tendinopathy in volleyball athletes.

